# Supplementary figures and images for: A background correction method to compensate illumination variation in hyperspectral imaging
Source: PLoS One. 2020 Mar 13;15(3):e0229502. doi: 10.1371/journal.pone.0229502 (PMC7069652; doi:10.1371/journal.pone.0229502)

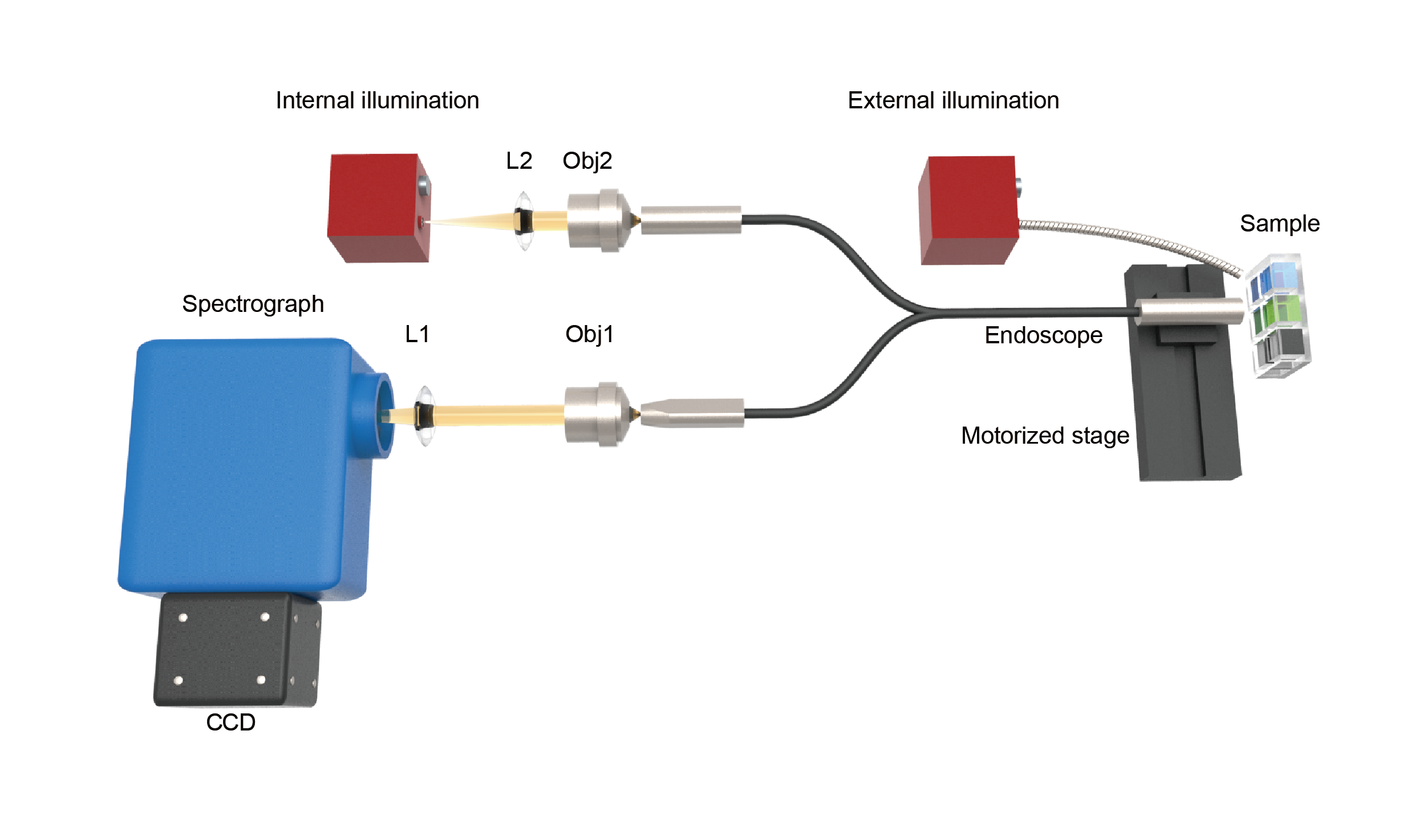

Supplement: S1 Fig — The system is assembled using a CE-marked endoscope with an imaging fibre bundle and an integrated illumination fibre. A sample is illuminated either by coupling a halogen light source to the illumination fibre (internal illumination method) or by directly illuminating via the fibre-coupled halogen light source (external illumination method). Hyperspectral data is acquired using a CCD coupled to the spectrograph. For line-scanning hyperspectral imaging, a motorized translational stage is exploited to control imaging position in these studies. Abbreviations: CCD, charge coupled device; L1–2, lens; Obj1–2, objective lens. (PNG) [file pone.0229502.s001.png]

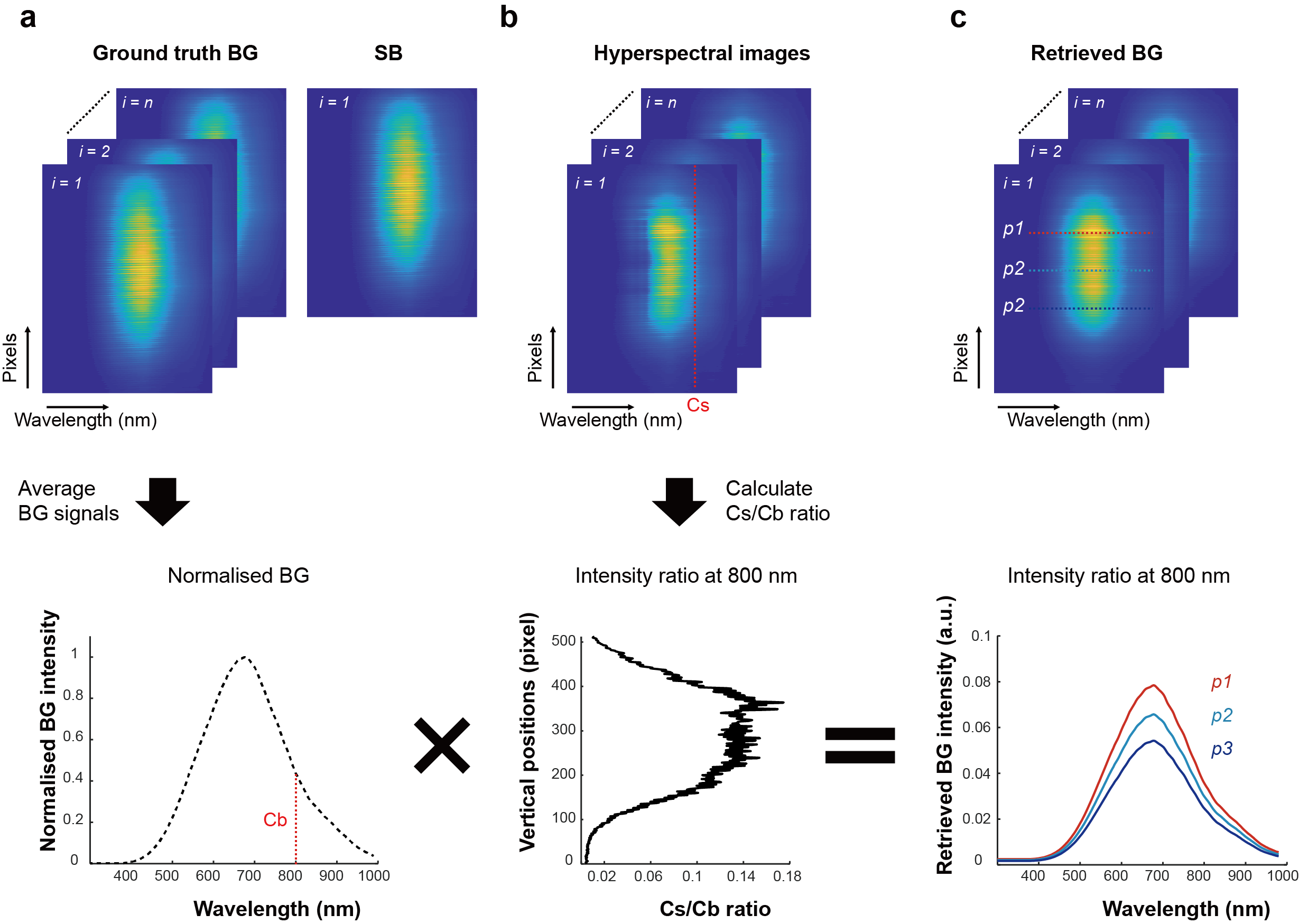

Supplement: S2 Fig — (a) GT was obtained by measuring a white reflectance target under the same position and illumination conditions as the sample measurement. From the GT, the normalised spectral profile of the background was calculated by averaging across all spatial locations within the hyperspectral image frame. One of GTs was used as SB. (b) To obtain RB, the intensity ratio (Cs/Cb) at 800 nm and the normalised spectral profile of the background was calculated. The intensity ratio of each vertical pixel was calculated by dividing intensity values of a sample spectral image (Cs, red dashed line) at 800 nm by the intensity value of normalised background signal at 800 nm (Cb). (c) The spectrum of the RB used for correction at a specific vertical pixel was determined by multiplying the normalised background to the intensity ratio value corresponding to the pixel. (PNG) [file pone.0229502.s002.png]

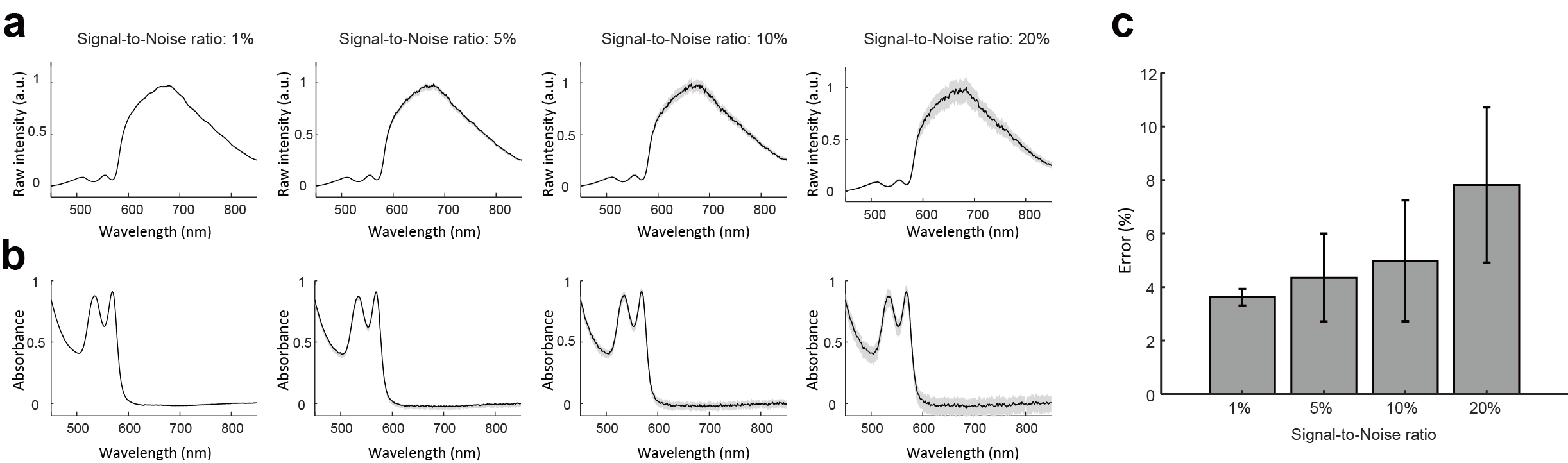

Supplement: S3 Fig — Influence of noise-to-signal ratio to the retrieved BG method (a) Simulation of raw spectral profiles of oxygenated blood with different signal-to-noise ratios (1%, 5%, 10%, and 20%). (b) Absorbance obtained using spectral signals in (a) and the retrieved BG method. Gray shaded area indicates the standard deviation. (c). Bar graphs show the average error percentages of absorbance at four different signal-to-noise ratios. Error bar indicates the standard deviation. (PNG) [file pone.0229502.s003.png]

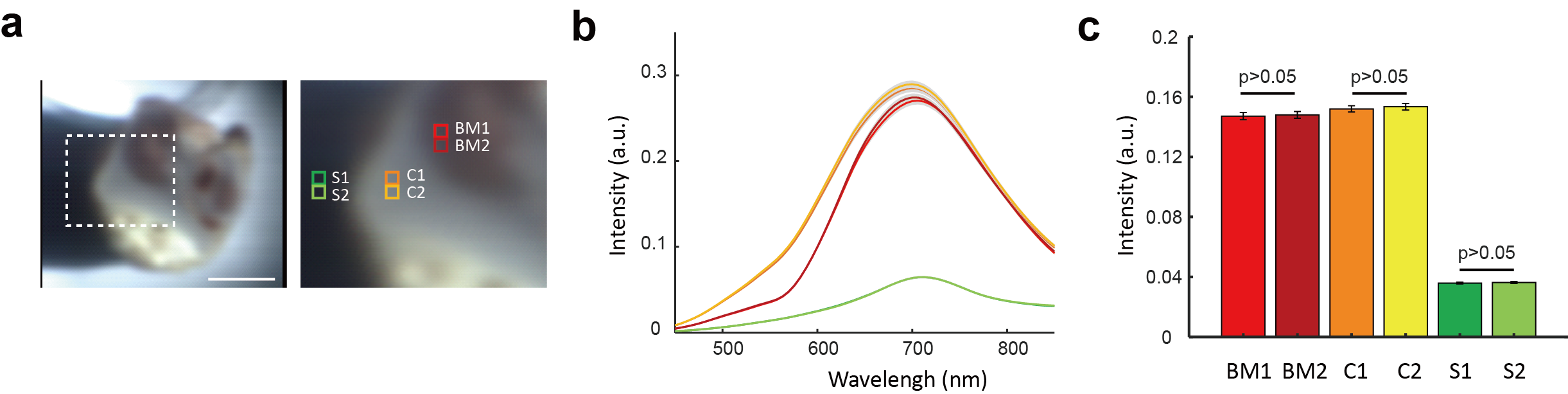

Supplement: S4 Fig — Investigation of intensity variation at 800 nm (a) Left: Synthetic RGB image of chicken tissue. Right: Magnified image of the dashed square shown in left figure. Scale bars: 1 cm (b) Average measured spectral profiles of the bone marrow (BM), compact bone (C), and shade (S) areas within solid squares shown in (a) were obtained. Gray shaded area indicates the standard deviation. (c). Bar graphs show average intensities of six regions shown in (a) were calculated. Error bar indicates the standard deviation. Statistical analysis was performed using Student t-test. (PNG) [file pone.0229502.s004.png]

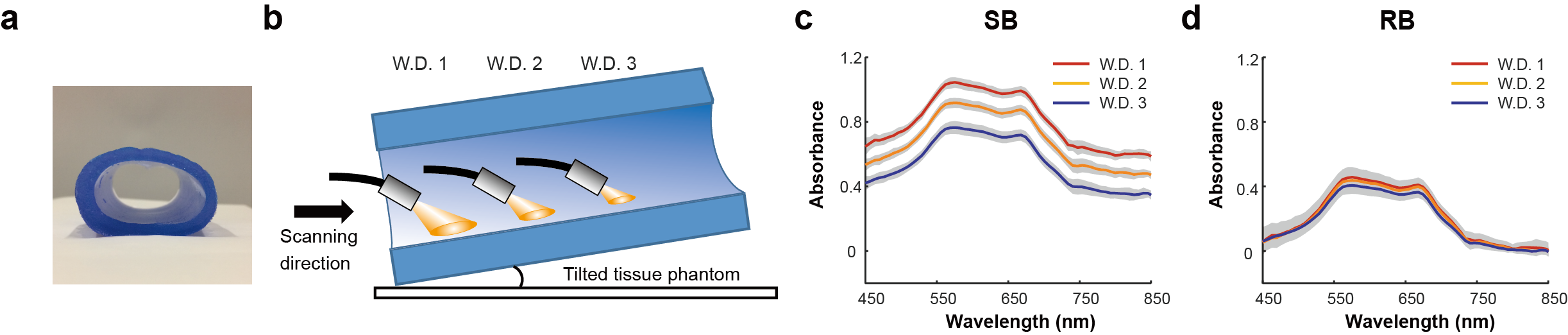

Supplement: S5 Fig — (a) Photograph of the tubular tissue-mimicking phantom with homogeneous methylene blue concentration. (b) Schematic of the experiment. Absorbance of the tissue-mimicking phantom at three working distances was obtained using SB (c) and RB methods (d). The solid line and the gray shaded area indicate average absorbance and standard deviation, respectively. (PNG) [file pone.0229502.s005.png]

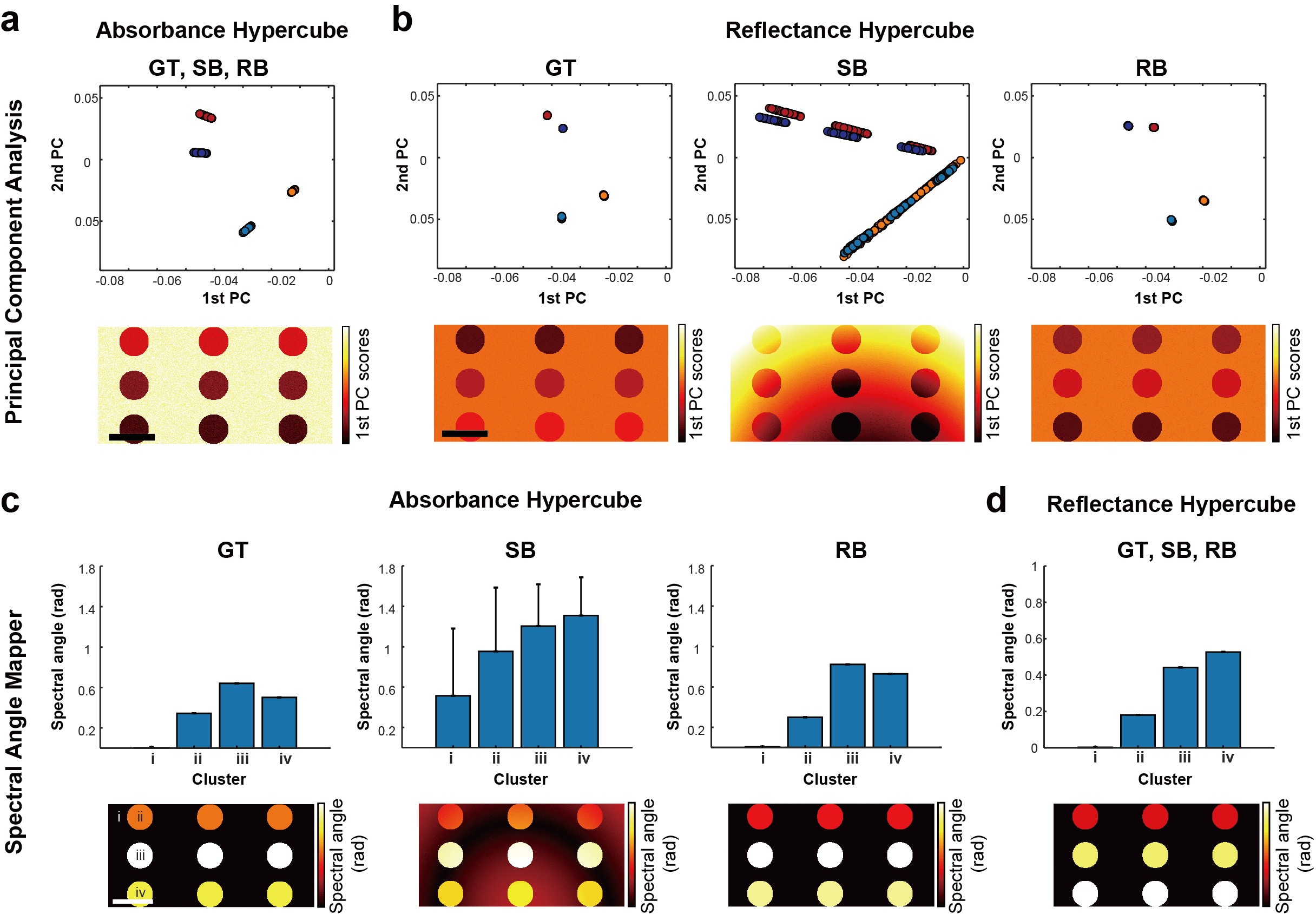

Supplement: S6 Fig — (a, b) Scatter plots of 2nd principal component (PC) versus 1st PC (top) and representative images of 1st PC scores (bottom) of absorbance and reflectance hypercubes, respectively. Scale bar: 100 pixels. (c, d) Bar graphs indicate mean and standard deviation (error bars) of angle values for each cluster shown in the bottom image (top) and 2D images of spectral angle values (bottom) of absorbance and reflectance hypercubes, respectively. SAM was performed using the average spectral profile of the cluster i of each hypercube. Scale bar is 100 pixels. (PNG) [file pone.0229502.s006.png]

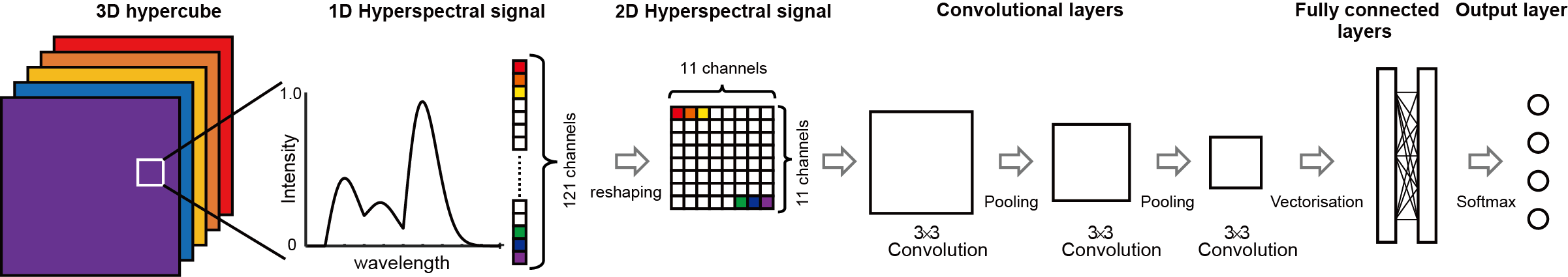

Supplement: S7 Fig — (PNG) [file pone.0229502.s007.png]
