# Supplementary material for: A background correction method to compensate illumination variation in hyperspectral imaging
Source: PLoS One. 2020 Mar 13;15(3):e0229502. doi: 10.1371/journal.pone.0229502 (PMC7069652; doi:10.1371/journal.pone.0229502)
Supplement: Attachment — Submitted filename: Response to Reviewers.docx [file pone.0229502.s008.docx]

**PONE-D-19-28216**

**A background correction method to compensate illumination variation in hyperspectral imaging**

We would like to take this opportunity to thank the Editor and the Reviewers for their careful assessment of our manuscript. We have provided a detailed response to each of the comments in the pages that follow and hope that our revised manuscript will have satisfactorily addressed the concerns that were raised.

**Editor comments:**

When submitting your revision, we need you to address these additional requirements.

Please ensure that your manuscript meets PLOS ONE's style requirements, including those for file naming. The PLOS ONE style templates can be found at

http://www.journals.plos.org/plosone/s/file?id=wjVg/PLOSOne_formatting_sample_main_body.pdf and http://www.journals.plos.org/plosone/s/file?id=ba62/PLOSOne_formatting_sample_title_authors_affiliations.pdf

🡪 We have carefully reviewed these files and believe that we have appropriately met the style requirements.

1. We note that you have stated that you will provide repository information for your data at acceptance. Should your manuscript be accepted for publication, we will hold it until you provide the relevant accession numbers or DOIs necessary to access your data. If you wish to make changes to your Data Availability statement, please describe these changes in your cover letter and we will update your Data Availability statement to reflect the information you provide.

🡪 We have added information about the data repository in the Materials and Methods section (<https://doi.org/10.17863/CAM.42338>). We will make this available as an open-access dataset once the manuscript is accepted.

2. Please do not include funding sources in the Acknowledgments or anywhere else in the manuscript file. Funding information should only be entered in the financial disclosure section of the submission system. https://journals.plos.org/plosone/s/submission-guidelines#loc-acknowledgments

🡪 According to the requirement, we have removed funding sources from the Acknowledgements.

3. Please amend the Methods section of your manuscript to include the source of the animal tissues

🡪 We included information about the source of the chicken tissue and mice blood in the ‘Materials and methods’ section. Food-grade chicken tissue was purchased from a local grocery market, and mice blood was obtained from the Biological Resources Unit of the Cancer Research UK Cambridge Institute.

**Reviewer #1:**

In this paper, the authors propose a simple method that is able to compensate the effects of illumination variations across a sample in hyperspectral imaging applications, using information about a specific spectral band in which the sample has approximately zero absorbance, and the spectral profile of the illumination source. This avoids the need for specialized illumination equipment.

The problem is important and well motivated (i.e. poorly compensated illumination affects subsequent image analysis tasks), and the method is simple but seems robust as long as the hypotheses are satisfied. Nonetheless, there are some points that could be improved.

We appreciate the feedback of the reviewer and we hope that the revisions made to the manuscript now improve the clarity of the description sufficiently.

The description of the proposed method in page 5 could be made clearer if supported by mathematical equations. More precisely, the effect of the illumination scaling alpha in equations 1 and 2, the relationship between C_b and C_s and the quantities introduced in equations 1 and 2, and the result of the proposed background correction could be shown in a few equations without substantially increasing the section's length.

🡪 We appreciate this thoughtful comment. We have added mathematical equations to make the description of the proposed method clear in the revised manuscript. These can now be found on pages 11 and 12 following the reformatting of the manuscript.

Also, note that alpha is used to denote both the illumination scaling and the spectral angle mapper result in equation 3, what could cause some confusion.

🡪 This is a very good point. As the reviewer suggested, we have changed the illumination scaling factor to be *ω* to remove possible confusion between the illumination scaling and the spectral angle mapper results.

A non-zero absorbance of the sample at the 800nm spectral band is shown to lead to a slight underestimation of the amplitude of the spectral samples. It also seems to be assumed that this absorbance is the same (i.e. theoretically zero) for all pixels in the sample. What effect would be observed in the proposed method if there are (e.g. small) spatial variations of this quantity? To which extent is this expected to occur in the application of interest? It would be interesting to see a discussion on this matter.

🡪 The comment of the reviewer is correct. The proposed method assumes that there is negligible or spatially consistent absorption at the normalisation wavelength, in this case at 800 nm. However, if there is a finite or spatially inhomogeneous absorption at 800 nm, the proposed method caused an error in calculating reflectance and absorbance. Therefore it is necessary to consider the properties of the sample being interrogated carefully when selecting the normalisation wavelength to ensure that one is confident of these criteria being met. We have added a paragraph on this in the discussion (pages 20-21)

- How is the pixel spectra reshaped in figure S.5 into a 2D signal?

🡪 For application into the convolutional neural network, we reshaped the 1D hyperspectral signal with 1 × 121 spectral channels into a 2D matrix with 11 × 11 spectral channels. 11 spectral channels of the hyperspectral signal become a row of the 2D reshaped matrix. We have added dimensional information and colours in the revised figure S.6 to better illustrate exactly how the matrix was reshaped.

**Reviewer #2:**

In first place I would like to thanks the authors. Despite the complexity of the topic of the paper, I found the paper well written and clear. Moreover, an interesting method to correct for illumination inhomogeneities directly form the measured sample is presented and tested in several ex-vivo conditions. Besides of this, here are some suggestions and comments that can enhance paper robustness.

We would, in turn, like to thank the reviewer for their kind comments and helpful suggestions, which we hope to have adequately addressed below.

Major comments:

1) Figure. 1: a confront is made between normalized and not normalized spectrum between the retrieved illumination and the measured illumination. It would be clearer if the comparison would be made on the same graph to show the consistency in between the spectral signals. I suggest to place both dashed and continue lines for the measured and the retrieved data, respectively. This comment applies to all the data where the differences between methods are minimal such as in the GT-RB comparisons in Figures 2-5.

🡪 We appreciate the reviewer’s careful comments. We have changed normalised and retrieved spectral profiles as dashed line to show the consistency among the spectral signals in the revised figures.

2) It is not clear to me why the author selects 800 nm as reference wavelength. Only reference 38 is given to support this choice. In my opinion a detailed explanation of the choice of 800 nm as reference wavelength must be provided. Taking under consideration the used sample and the measurements performed. This will help also to understand how this method could be used for different samples where the wavelengths that must be selected would be different.

🡪 There are two reasons that we selected 800 nm as the normalisation wavelength. First, most biological tissues have negligible absorbance when illuminated using near-infrared light. We have added a further recent reference about the scattering and absorption properties in biological tissue in the revised manuscript. Second, there is an isosbestic absorption at 800 nm in oxygenated and deoxygenated blood, so if there were any impact of the absorption on our experiments, it would not be affected by the oxygenation status of the blood and would still allow us to retrieve that biological information using the data in the visible wavelengths. We have added this additional point in the revised manuscript.

3) If I did not misunderstand the text, the information at 800 nm is completely lost no matter what the sample. If it is the case add a statement on this in the text

🡪 The reviewer’s comment is correct. The proposed method normalises the spectrum based on 800 nm; thus, the information at 800 nm is lost. We have now discussed this limitation in the revised manuscript. As there are several isosbestic points of haemoglobin in the visible range, it should not be a problem in our study. However, if the information at 800 nm is important (e.g. NIR dyes), then another normalisation wavelength should be selected.

4) In Fig 5 all the information after 750 nm is cut. Please insert all the spectral information to confront these spectra with all the others.

🡪 We have revised the figure 5 according to the reviewer’s comment.

5) Which is the influence of using 800 nm to correct these images in terms of artifacts? I propose a study of the variation of the intensity at very small area where the illumination light can be considered perfectly homogenous and independent from the illumination conditions in the case of the chicken breast where the sample is not perfectly homogeneous in reflectance. This validation will grant more robustness to the overall article. Is not clear to me the variations of spectral information at 800 nm in its absolute value. If the differences are considerable the method proposed will produce unpredictable artifacts. Maybe the Root Mean Square value could be calculated in between standard deviations calculated from the data collected on different areas (marrow and compact bone). In this way the effect of the 800 nm choice can be estimated.

🡪 We appreciate the reviewer’s constructive comment. We have analysed raw intensities at 800 nm of two very small areas (4 × 4 pixels) within the bone marrow, compact bone, and shadowed area. There was no significant difference in raw intensities of each tissue type, which means there is a minimum influence of intensity noise at 800 nm to the proposed method. We have added a supplementary figure (S4) and updated the results in the revised manuscript.

6) “Despite these limitations, the proposed background correction method allows for accurate and consistent measurement of HSI data, regardless of illumination methods and optical power distribution.”

This is partially true as one of the assumptions is that every spectral channel must be acquired in the same illumination condition. In other words that means a good superposition between spectral channels. That is the case of most of the applications when the spectral sampling is performed in the detection, but it is not true in the case that several light sources are used instead.

🡪 The reviewer’s comment is correct. The proposed method can’t be used if there is spectrally inhomogeneous illumination condition. We have added this to Discussion.

7) In this case an a-priori study of the sample is necessary at least to select the right wavelength. I suggest to add a statement that underline this. In fact, if the sample is highly etherogenous in its composition, with high variations for all the wavelength in the spectral range this method cannot be blind as it will gives an unpredictable result in terms of spectral information.

🡪 We agree with the reviewer’s comment. This method cannot be used if we don’t have information that there is no significant absorbance at 800 nm. Thus we have revised the manuscript to make it clear that this method requires prior knowledge about the absorbance of the sample at the normalisation wavelength.

Minor comments:

8)The “hypercube is a n-dimensional volume of data for a 3D hyperpestral cube, in my advise it would be better datacube”

🡪 In general, hyperspectral imaging data is called as ‘hypercube’. Therefore we prefer to retain the ‘hypercube’ terminology to make consistent with the other papers in the hyperspectral imaging research field.

**Reviewer #3:**

This manuscript proposed a background correction method to compensate for the variations in surface morphology or light power distribution at the sample. The proposed background correction method enables the estimation of optical characteristics of illumination at the sample, which is a major advance in hyperspectral endoscopy systems. The authors experimentally demonstrated the feasibility of the proposed background correction method using hyperspectral imaging data acquired via a hyperspectral endoscopy system from different samples. The proposed method and experiment results are useful in further exploitation of hyperspectral imaging endoscopy systems in practical clinical applications, the manuscript can be accepted for publication.

We would like to thank the reviewer for their time in reviewing our manuscript.

A few questions that should be addressed:

1. Where is the multiplicative factor alpha in eq. (1) and constant in eq. (2) ?

🡪 We have added two further equations (eq. 4 and 5) to make this point clear in the revised manuscript.

2. In the Result section P5, line 10. Did you multiply the normalized spectrum of the light source with the intensity ratio between the measured spectral profiles of the light source and the sample or with the intensity ratio between the normalized spectral profiles of light source and sample?

🡪 We have added two further equations (eq. 6 and 7) for a better description of the proposed method in the revised manuscript.

3. The wavelength 800 nm was selected as the band with low absorption to verify the feasibility of the proposed method. The question is, if the absorption of a certain tissue is not that small at 800nm, does the method still work? If not, how to solve this problem?

🡪 If there is finite absorption at 800 nm, the proposed method would lead to a scale shift in the reflectance and absorbance, although in the examples we use in the manuscript, does not change the spectral profile. We have now discussed this more extensively in the revised manuscript.

4. In figure 5a, there is no much difference in darkness between RGB images of GT and SB? In contrast, GT and SB looks similar.

🡪 The difference in the synthetic RGB images arises because GT shows homogeneous brightness, but SB has bright regions on the right side of the sample and gets darker toward the left. This is because SB does not include information about illumination intensity.

5. In figure 5a, six colored rectangular boxes are obscure.

🡪 We have changed the blue colour squares as green colours and increased line widths to make the figure clear.

**Reviewer #4:**

The article by Yoon et al. presents a relatively simple approach to correct intensity variations in sample illumination in HSI applications. Overall, the presented approach is scientifically sound and offers an easy correction option with some assumptions. However, the simplicity of the approach means it is not innovative in general, but rather as an application to a specific field.

We thank the reviewer for their positive appraisal of the manuscript and hope that we have adequately addressed their concerns in the response below.

There a few major points to be addressed:

1) Sensitivity to noise – a method calculating a scaling coefficient only using a single wavelength will be more susceptible to noise over methods using multiple. Since most of the work is using some sort of simulation, it would be interesting to see the influence of varying levels of noise on the retrieved signal. Please add such calculations.

🡪 We appreciate the reviewer’s insightful comment. The noise levels in spectral profiles would affect the accuracy of the proposed method due to using a single normalisation wavelength. As reviewer suggested, we performed simulation work to check the influence of noise levels to the accuracy. We added four different noise levels (1%, 5%, 10%, and 20%) to the spectral profile of oxygenated blood, then calculated absorbance using the simulated spectral profiles and the proposed method (Supplementary Fig. S7a,b). Supplementary Fig. S7c shows that the error levels are 3.62 ± 0.31%, 4.35 ± 1.64, 4.98 ± 2.26%, and 7.81 ± 2.90% as increasing a noise level from 1% to 20%, respectively. This means that the accuracy of the proposed method will indeed decrease in the scenario of very noisy spectral data. We have added this result and discussion in the revised manuscript.

2) Compare to other baseline correction methods (e.g. independent component analysis, fitting) and show the benefits of this approach. It may be in the lack of optimization parameters and speed.

🡪 The baseline correction methods (ICA and fitting) the reviewer mentions are mainly used to separate source and target signals. However, our motivation is to estimate illumination power from unknown illumination conditions. Thus, we used a normalised spectral profile and ratio between the normalised spectral profile and measured raw sample signals at the normalisation wavelength. Therefore, we compared our approach with other methods that are used for illumination power correction (as described in the Introduction) rather than the suggested baseline correction methods.

3) The machine learning part in general is valid, however, a small detail would make a big difference. The authors did a min/max scaling for the SB dataset, while anyone doing spectroscopy and machine learning would also remove the baseline in any way before doing that. Subtract minimum value or a linear fit (or any other baseline remocal) for SB and then do classification for KNN and SVM.

🡪 As the review mentions, it is usual to remove baseline in some way before applying machine learning methods. For machine learning, all our synthetic hypercubes (GT, SB, and RB dataset) were subjected to min-max normalisation, which means every dataset had their minimum value subtracted.

A smaller point is to slightly clear out the introduction about absorbance and reflectance with scaling factor, since in a proper spectroscopic experiment the scaling would also affect background and be removed in the proportion of I/I0. The lack of proper background in these experiments is the problem.

🡪 Reviewer’s comment is correct. The proper spectroscopic experiment measures precise spectral profiles of both the background and sample and then calculates reflectance and absorbance by using the proportion of I/I0. In this calculation, the scaling is removed. However, the precise challenge that we address in this paper is that in many biomedical spectroscopic experiments it is not possible to measure an accurate background signal, for example, during clinical endoscopic imaging conditions; there are different scaling factors between spectral profiles in the background and sample. It is precisely to overcome this limitation that we proposed the method that enables the estimation of background illumination power based on the ratio between a normalised spectrum of the light source and measured sample signal at the normalisation wavelength. We have added equations 3-6 to make the concept of the proposed method clear.

There are also a few small language inaccuracies here and there.

🡪 We hope that we have spotted and corrected them all in the revised manuscript.
